# Cold War, Deadly Fevers: Malaria Eradication in Mexico, 1955–1975 

**DOI:** 10.3201/eid1403.071564

**Published:** 2008-03

**Authors:** Paul Arguin

**Affiliations:** * Centers for Disease Control and Prevention, Atlanta, Georgia, USA

Marcos Cueto is a medical historian who describes the details of malaria eradication efforts in Mexico in the context of the Cold War era authoritarianism. His approach works overall, but occasionally he overreaches. 

Mr. Cueto asserts that the politics of the time allowed the medical community to be similarly authoritarian in forcing malaria eradication to be the accepted strategy. He states that the political climate relied on fear-based tactics of spreading anxiety about the communist threat and that similar strategies were used to gain public support for the malaria eradication effort, incorporating military jargon such as “enemy” mosquitoes and “campaigns” against disease into the public health lexicon. These campaigns included a propaganda arm in which pop stars became champions for the cause, to make the public sympathetic to their efforts. At one point, the author likens the strategy of screening persons for asymptomatic malaria parasitemia to the 1950s McCarthy-style witch hunts for hidden communists—stretching the analogy beyond tolerable limits.

A recurring theme in the book is Mr. Cueto’s skepticism of new technologies, especially those introduced by other (non-Mexican) national or international organizations. He disparages the adoption of chloroquine, DDT, and smear microscopy as “magic bullet” strategies, claiming they distracted from rather than enhanced control efforts. He suggests that current efforts, which tout the use of long-lasting insecticide-treated bed nets and artemisinin-based combination therapy, are similarly flawed. He never really articulates proven alternatives to the adoption of new technologies, but he vaguely suggests relying on community-based broad public health programs at the grassroots level.

I found the tone of the book a bit cynical and fatalistic. I got the impression that Mr. Cueto believes that all individuals and organizations attempting malaria eradication were doing so not for its own sake but rather as a front for other agendas such as centralizing national power in Mexico, furthering the international interests of capitalist countries, or personal glory. The author uses the fact that the eradication effort failed to support his contention that it was probably a bad idea in the first place.

Fast forward to today when there is a renewed interest in malaria control efforts. Witness the Global Fund to Fight AIDS, Tuberculosis, and Malaria; the Bill & Melinda Gates Foundation; the President’s Malaria Initiative; and pop stars such as Bono championing the cause, including the possibility of eradication. Sounds familiar. In light of the current progress of malaria control efforts in Mexico, where most states are now malaria free and the total number of cases has been steadily decreasing, Mexico is well on its way to achieving those original eradication goals. Thus, whether you agree with the author’s politics or not, if you are considering getting into the business of malaria eradication, you could benefit by reviewing this very detailed historical account of a malaria eradication effort that was unsuccessful.

